# Bis(methacrylato-κ*O*)bis­(2,4,6-trimethyl­pyridine-κ*N*)copper(II)

**DOI:** 10.1107/S1600536812009919

**Published:** 2012-03-24

**Authors:** Ejaz, Islam Ullah Khan, Alina Murtaza, William T. A. Harrison

**Affiliations:** a Materials Chemistry Laboratry, Department of Chemistry, GC University, Lahore 54000, Pakistan; b Department of Chemistry, University of Aberdeen, Meston Walk, Aberdeen AB24 3UE, Scotland

## Abstract

In the monomeric title complex, [Cu(C_4_H_5_O_2_)_2_(C_8_H_11_N)_2_], the Cu^II^ atom lies on a centre of inversion. Its coordination by two substituted pyridine ligands and two carboxyl­ate anions leads to a slightly distorted *trans*-CuN_2_O_2_ square-planar geometry. The dihedral angle between the mean planes of the pyridine (py) ring and the carboxyl­ate group is 74.71 (7)°. The dihedral angles between the planar CuN_2_O_2_ core and the py ring and carboxyl­ate plane are 67.72 (5) and 89.95 (5)°, respectively. Based on the refined C=C and C—C bond lengths, the terminal =CH_2_ and –CH_3_ groups of the carboxyl­ate anion may be disordered, but the disorder could not be resolved in the present experiment. Several intra­molecular C—H⋯O inter­actions occur. In the crystal, mol­ecules are linked by weak C—H⋯O hydrogen bonds, generating chains propagating in [100].

## Related literature

For the crystal structures of related monomeric complexes containing a *trans*-CuN_2_O_2_ core, see: Borel *et al.* (1981 ▶); Heimer & Ahmed (1982 ▶); Jedrzejas *et al.* (1994 ▶).
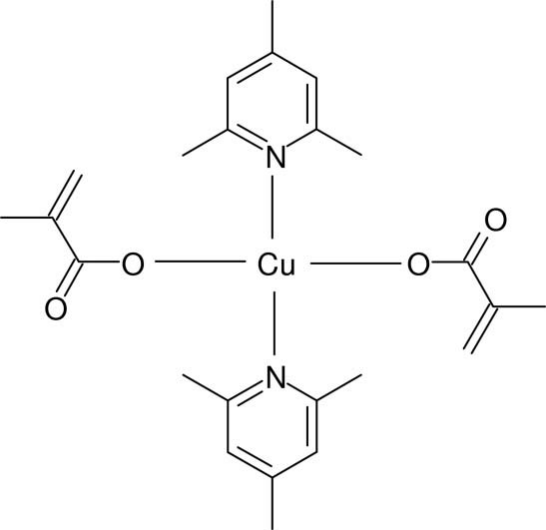


## Experimental

### 

#### Crystal data

[Cu(C_4_H_5_O_2_)_2_(C_8_H_11_N)_2_]
*M* *_r_* = 476.06
Monoclinic, 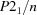

*a* = 8.2295 (2) Å
*b* = 17.0921 (6) Å
*c* = 9.1683 (3) Å
β = 109.220 (1)°
*V* = 1217.73 (7) Å^3^
*Z* = 2
Mo *K*α radiation
μ = 0.93 mm^−1^
*T* = 296 K
0.08 × 0.06 × 0.06 mm

#### Data collection

Bruker APEXII CCD diffractometer
Absorption correction: multi-scan (*SADABS*; Bruker, 2007 ▶) *T*_min_ = 0.930, *T*_max_ = 0.947
11795 measured reflections
3017 independent reflections
2439 reflections with *I* > 2σ(*I*)
*R*_int_ = 0.030

#### Refinement

*R*[*F* ^2^ > 2σ(*F* ^2^)] = 0.032
*wR*(*F* ^2^) = 0.098
*S* = 1.05
3017 reflections
146 parameters
H-atom parameters constrained
Δρ_max_ = 0.24 e Å^−3^
Δρ_min_ = −0.21 e Å^−3^

### 

Data collection: *APEX2* (Bruker, 2007 ▶); cell refinement: *SAINT* (Bruker, 2007 ▶); data reduction: *SAINT*; program(s) used to solve structure: *SHELXS97* (Sheldrick, 2008 ▶); program(s) used to refine structure: *SHELXL97* (Sheldrick, 2008 ▶); molecular graphics: *ORTEP-3* (Farrugia, 1997 ▶); software used to prepare material for publication: *SHELXL97*.

## Supplementary Material

Crystal structure: contains datablock(s) I, global. DOI: 10.1107/S1600536812009919/rn2095sup1.cif

Structure factors: contains datablock(s) I. DOI: 10.1107/S1600536812009919/rn2095Isup2.hkl

Additional supplementary materials:  crystallographic information; 3D view; checkCIF report

## Figures and Tables

**Table:** 

Cu1—O2	1.9406 (12)
Cu1—N1	2.0404 (14)

**Table:** 

O2—Cu1—N1	91.73 (6)

**Table 2:** Hydrogen-bond geometry (Å, °)

*D*—H⋯*A*	*D*—H	H⋯*A*	*D*⋯*A*	*D*—H⋯*A*
C4—H4⋯O1^i^	0.93	2.45	3.338 (2)	161
C6—H6*A*⋯O1	0.96	2.49	3.369 (3)	153
C6—H6*C*⋯O2^ii^	0.96	2.48	3.139 (3)	126
C8—H8*A*⋯O1^ii^	0.96	2.49	3.357 (3)	150
C8—H8*C*⋯O2	0.96	2.51	3.124 (2)	122

Symmetry codes: (i) 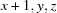; (ii) 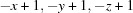.

**Fig. 1.**
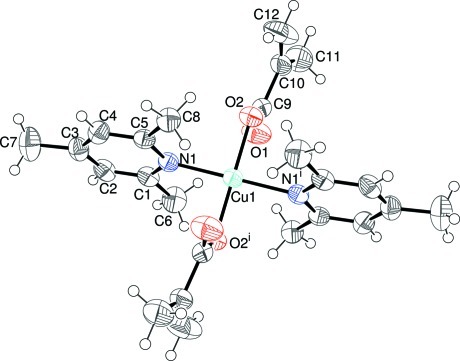
The molecular structure of (I) showing 50% displacement ellipsoids. Symmetry code: (i) 1–x, 1–y, 1–z.

**Fig. 2.**
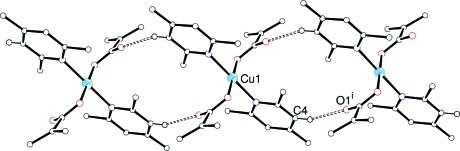
Fragment of a [100] chain of complex molecules linked by C—H···O hydrogen bonds (double dashed lines). Symmetry code: (i) 1+x, y, z.

## supplementary crystallographic information

### Comment

The title compound, (I), is a centrosymmetric neutral monomeric copper(II) complex (Fig. 1). Related structures containing a copper(II) ion bonded to a pair of susbtituted pyridine ligands and a pair of monodentate carboxylate anions have been described previously (Borel *et al.*, 1981; Heimer & Ahmed, 1982; Jedrzejas *et al.*, 1994).

The Cu ion in (I) lies on an inversion centre, resulting in a slightly distorted trans-CuN_2_O_2_ square planar geometry for the metal ion (Table 1). If a very long contact between Cu1 and O1 [2.8229 (17)Å] is considered to have any significance as a bond, a grossly disorted trans-CuN_2_O_4_ octahedron results. The mean planes of the pyridine ring (r.m.s. deviation = 0.0099Å) and the carboxylate group (r.m.s. deviation = 0.0003Å) are roughly perpendicular [dihedral angle = 74.71 (7)°], which presumably minimises steric interactions between the ligands. The dihedral angles between the planar CuN_2_O_2_ core and the py ring and carboxylate plane are are 67.72 (5) and 89.95 (5)°, respectively. The Cu ion is displaced by 0.256 (3)Å from the py ring plane and by 0.252 (3)Å from the carboxylate plane. The carboxylate C—O bond lengths of 1.222 (2)Å for O1 and 1.276 (2)Å for O2 suggest the presence of relatively localised single and double bonds in the anion.

The terminal CH_2_ and CH_3_ groups of the carboxylate anion are probably disordered: the nominal C10—C11 single bond is short [1.422 (4)Å] and the nominal C10=C12 double bond is long [1.378 (4)Å]. This may also correlate with the Hirshfeld rigid bond alert for the C10—C12 bond. The presumed disorder could not be resolved in the present experiment. Several intramolecular C—H···O interactions occur (Table 1). In the crystal, the molecules are linked by C—H···O hydrogen bonds to generate chains in the [100] direction.

In trans-bis(acetato-O)bis(4-methyl pyridine-N)copper(II) (Jedrzejas *et al.*, 1994), (II), the dihedral angle between the ligands is 78.2° (s.u. not stated), and the dihedral angle between the py ring and the CuN_2_O_2_ plane is 31.6°. The uncoordinated Cu···O separation of 2.623 (4)Å in (II) is significantly shorter than that seen in (I). However, it is notable that the carboxylate C—O bonds lengths in (II) [1.227 (7) and 1.279 (6)Å] are almost identical to those seen here.

### Experimental

Copper sulfate (0.16 g, 1.0 mmol) was dissolved in methanol (20 ml). Then, 2,4,6-trimethyl pyridine (0.264 ml, 2.0 mmol) was added to this solution, which turned green. This reaction mixture was refluxed for 30 minutes followed by addition of methacrylic acid (0.169 ml, 2.0 mmol), at which point the solution remained green. After refluxing for one hour, the solution was filtered and kept for a few days. Blue-green blocks of (I) were obtained from filtrate by slow evaporation.

### Refinement

Attempts were made to represent the disordered C11 (nominal CH_2_ group) and C12 (nominal CH_3_ group) atoms with a double-site model, but the refinement was unstable. The hydrogen atoms were placed in calculated positions (C—H = 0.93–0.96Å) and refined as riding atoms with U_iso_(H) = 1.2U_eq_(C) or 1.5U_eq_(methyl C). The methyl groups were allowed to rotate, but not to tip, to best fit the electron density.

### Crystal data

**Table:**

[Cu(C_4_H_5_O_2_)_2_(C_8_H_11_N)_2_]	*F*(000) = 502
*M**_r_* = 476.06	*D*_x_ = 1.298 Mg m^−^^3^
Monoclinic, *P*2_1_/*n*	Mo *K*α radiation, λ = 0.71073 Å
Hall symbol: -P 2yn	Cell parameters from 3020 reflections
*a* = 8.2295 (2) Å	θ = 2.4–28.3°
*b* = 17.0921 (6) Å	µ = 0.93 mm^−^^1^
*c* = 9.1683 (3) Å	*T* = 296 K
β = 109.220 (1)°	Block, blue-green
*V* = 1217.73 (7) Å^3^	0.08 × 0.06 × 0.06 mm
*Z* = 2	

### Data collection

**Table:**

Bruker APEXII CCD diffractometer	3017 independent reflections
Radiation source: fine-focus sealed tube	2439 reflections with *I* > 2σ(*I*)
Graphite monochromator	*R*_int_ = 0.030
ω scans	θ_max_ = 28.3°, θ_min_ = 2.9°
Absorption correction: multi-scan (*SADABS*; Bruker, 2007)	*h* = −10→10
*T*_min_ = 0.930, *T*_max_ = 0.947	*k* = −22→21
11795 measured reflections	*l* = −12→12

### Refinement

**Table:**

Refinement on *F*^2^	Primary atom site location: structure-invariant direct methods
Least-squares matrix: full	Secondary atom site location: difference Fourier map
*R*[*F*^2^ > 2σ(*F*^2^)] = 0.032	Hydrogen site location: inferred from neighbouring sites
*wR*(*F*^2^) = 0.098	H-atom parameters constrained
*S* = 1.05	*w* = 1/[σ^2^(*F*_o_^2^) + (0.0554*P*)^2^ + 0.2257*P*] where *P* = (*F*_o_^2^ + 2*F*_c_^2^)/3
3017 reflections	(Δ/σ)_max_ = 0.001
146 parameters	Δρ_max_ = 0.24 e Å^−^^3^
0 restraints	Δρ_min_ = −0.21 e Å^−^^3^

### Special details

**Table:**

Geometry. All esds (except the esd in the dihedral angle between two l.s. planes) are estimated using the full covariance matrix. The cell esds are taken into account individually in the estimation of esds in distances, angles and torsion angles; correlations between esds in cell parameters are only used when they are defined by crystal symmetry. An approximate (isotropic) treatment of cell esds is used for estimating esds involving l.s. planes.
Refinement. Refinement of F^2^ against ALL reflections. The weighted R-factor wR and goodness of fit S are based on F^2^, conventional R-factors R are based on F, with F set to zero for negative F^2^. The threshold expression of F^2^ > 2sigma(F^2^) is used only for calculating R-factors(gt) etc. and is not relevant to the choice of reflections for refinement. R-factors based on F^2^ are statistically about twice as large as those based on F, and R- factors based on ALL data will be even larger.

### Fractional atomic coordinates and isotropic or equivalent isotropic displacement parameters (Å^2^)

**Table:**

	*x*	*y*	*z*	*U*_iso_*/*U*_eq_	
Cu1	0.5000	0.5000	0.5000	0.03574 (11)	
N1	0.66520 (18)	0.59275 (8)	0.54344 (15)	0.0370 (3)	
C1	0.6584 (2)	0.64975 (10)	0.64342 (19)	0.0418 (4)	
C2	0.7823 (2)	0.70758 (11)	0.6865 (2)	0.0468 (4)	
H2	0.7740	0.7465	0.7546	0.056*	
C5	0.7941 (2)	0.59402 (10)	0.48251 (18)	0.0381 (3)	
C9	0.2632 (2)	0.57402 (11)	0.2626 (2)	0.0437 (4)	
C3	0.9184 (2)	0.70825 (11)	0.6297 (2)	0.0481 (4)	
C8	0.7956 (2)	0.53238 (12)	0.3681 (2)	0.0473 (4)	
H8A	0.7929	0.4817	0.4124	0.071*	
H8B	0.8984	0.5372	0.3410	0.071*	
H8C	0.6967	0.5385	0.2772	0.071*	
C4	0.9208 (2)	0.65059 (11)	0.5248 (2)	0.0456 (4)	
H4	1.0089	0.6499	0.4821	0.055*	
C6	0.5137 (3)	0.64778 (13)	0.7083 (3)	0.0599 (5)	
H6A	0.4061	0.6456	0.6253	0.090*	
H6B	0.5173	0.6940	0.7686	0.090*	
H6C	0.5250	0.6024	0.7725	0.090*	
C10	0.1618 (3)	0.59108 (12)	0.0974 (2)	0.0568 (5)	
C7	1.0593 (3)	0.76821 (15)	0.6826 (4)	0.0776 (7)	
H7A	1.0113	0.8174	0.6986	0.116*	
H7B	1.1137	0.7743	0.6053	0.116*	
H7C	1.1429	0.7513	0.7776	0.116*	
C11	−0.0020 (4)	0.62688 (19)	0.0676 (4)	0.0899 (9)	
H11A	−0.0557	0.6336	−0.0418	0.135*	
H11B	0.0123	0.6770	0.1175	0.135*	
H11C	−0.0730	0.5942	0.1069	0.135*	
C12	0.2280 (5)	0.57196 (18)	−0.0179 (3)	0.0934 (9)	
H12A	0.1650	0.5824	−0.1205	0.112*	
H12B	0.3358	0.5486	0.0070	0.112*	
O1	0.2181 (2)	0.60068 (11)	0.36686 (17)	0.0675 (4)	
O2	0.39586 (16)	0.53088 (9)	0.28566 (14)	0.0477 (3)	

### Atomic displacement parameters (Å^2^)

**Table:**

	*U*^11^	*U*^22^	*U*^33^	*U*^12^	*U*^13^	*U*^23^
Cu1	0.03618 (17)	0.03855 (17)	0.03315 (16)	0.00306 (11)	0.01229 (11)	−0.00093 (11)
N1	0.0376 (7)	0.0391 (7)	0.0338 (7)	0.0041 (6)	0.0111 (5)	−0.0006 (5)
C1	0.0445 (9)	0.0422 (9)	0.0369 (8)	0.0073 (7)	0.0111 (7)	−0.0017 (7)
C2	0.0508 (10)	0.0402 (9)	0.0429 (9)	0.0065 (8)	0.0067 (8)	−0.0061 (7)
C5	0.0382 (8)	0.0397 (8)	0.0357 (8)	0.0064 (7)	0.0110 (6)	0.0032 (6)
C9	0.0429 (9)	0.0445 (9)	0.0413 (9)	−0.0048 (7)	0.0105 (7)	0.0056 (7)
C3	0.0410 (9)	0.0397 (9)	0.0554 (10)	0.0020 (7)	0.0048 (8)	0.0008 (8)
C8	0.0463 (10)	0.0512 (10)	0.0501 (10)	0.0021 (8)	0.0234 (8)	−0.0064 (9)
C4	0.0385 (9)	0.0440 (9)	0.0541 (10)	0.0036 (7)	0.0148 (8)	0.0042 (8)
C6	0.0649 (12)	0.0605 (12)	0.0639 (12)	0.0007 (10)	0.0341 (10)	−0.0178 (10)
C10	0.0626 (12)	0.0492 (11)	0.0448 (10)	−0.0087 (9)	−0.0011 (9)	0.0090 (8)
C7	0.0593 (14)	0.0602 (14)	0.105 (2)	−0.0122 (11)	0.0164 (13)	−0.0197 (14)
C11	0.0769 (17)	0.089 (2)	0.0811 (18)	0.0016 (15)	−0.0048 (13)	0.0307 (16)
C12	0.131 (2)	0.101 (2)	0.0391 (11)	0.0127 (19)	0.0155 (13)	−0.0022 (13)
O1	0.0591 (9)	0.0941 (12)	0.0519 (8)	0.0208 (8)	0.0218 (7)	0.0076 (8)
O2	0.0486 (7)	0.0534 (7)	0.0385 (6)	0.0038 (6)	0.0109 (5)	0.0035 (6)

### Geometric parameters (Å, º)

**Table:**

Cu1—O2	1.9406 (12)	C8—H8A	0.9600
Cu1—O2^i^	1.9406 (12)	C8—H8B	0.9600
Cu1—N1^i^	2.0404 (14)	C8—H8C	0.9600
Cu1—N1	2.0404 (14)	C4—H4	0.9300
N1—C1	1.352 (2)	C6—H6A	0.9600
N1—C5	1.352 (2)	C6—H6B	0.9600
C1—C2	1.381 (3)	C6—H6C	0.9600
C1—C6	1.496 (3)	C10—C12	1.378 (4)
C2—C3	1.382 (3)	C10—C11	1.422 (4)
C2—H2	0.9300	C7—H7A	0.9600
C5—C4	1.381 (3)	C7—H7B	0.9600
C5—C8	1.490 (2)	C7—H7C	0.9600
C9—O1	1.222 (2)	C11—H11A	0.9600
C9—O2	1.276 (2)	C11—H11B	0.9600
C9—C10	1.498 (3)	C11—H11C	0.9600
C3—C4	1.382 (3)	C12—H12A	0.9300
C3—C7	1.503 (3)	C12—H12B	0.9300
			
O2—Cu1—O2^i^	180.0	H8B—C8—H8C	109.5
O2—Cu1—N1^i^	88.27 (6)	C5—C4—C3	120.81 (17)
O2^i^—Cu1—N1^i^	91.73 (6)	C5—C4—H4	119.6
O2—Cu1—N1	91.73 (6)	C3—C4—H4	119.6
O2^i^—Cu1—N1	88.27 (6)	C1—C6—H6A	109.5
N1^i^—Cu1—N1	180.0	C1—C6—H6B	109.5
C1—N1—C5	118.81 (15)	H6A—C6—H6B	109.5
C1—N1—Cu1	121.25 (11)	C1—C6—H6C	109.5
C5—N1—Cu1	119.66 (11)	H6A—C6—H6C	109.5
N1—C1—C2	121.22 (16)	H6B—C6—H6C	109.5
N1—C1—C6	118.00 (16)	C12—C10—C11	122.9 (2)
C2—C1—C6	120.77 (16)	C12—C10—C9	120.0 (2)
C1—C2—C3	120.78 (17)	C11—C10—C9	117.1 (2)
C1—C2—H2	119.6	C3—C7—H7A	109.5
C3—C2—H2	119.6	C3—C7—H7B	109.5
N1—C5—C4	121.21 (15)	H7A—C7—H7B	109.5
N1—C5—C8	117.99 (15)	C3—C7—H7C	109.5
C4—C5—C8	120.81 (15)	H7A—C7—H7C	109.5
O1—C9—O2	123.32 (17)	H7B—C7—H7C	109.5
O1—C9—C10	120.46 (18)	C10—C11—H11A	109.5
O2—C9—C10	116.23 (17)	C10—C11—H11B	109.5
C4—C3—C2	117.10 (17)	H11A—C11—H11B	109.5
C4—C3—C7	121.63 (19)	C10—C11—H11C	109.5
C2—C3—C7	121.26 (19)	H11A—C11—H11C	109.5
C5—C8—H8A	109.5	H11B—C11—H11C	109.5
C5—C8—H8B	109.5	C10—C12—H12A	120.0
H8A—C8—H8B	109.5	C10—C12—H12B	120.0
C5—C8—H8C	109.5	H12A—C12—H12B	120.0
H8A—C8—H8C	109.5	C9—O2—Cu1	113.19 (11)
			
O2—Cu1—N1—C1	115.81 (13)	C1—C2—C3—C4	2.3 (3)
O2^i^—Cu1—N1—C1	−64.19 (13)	C1—C2—C3—C7	−176.5 (2)
O2—Cu1—N1—C5	−70.31 (12)	N1—C5—C4—C3	−0.7 (3)
O2^i^—Cu1—N1—C5	109.69 (12)	C8—C5—C4—C3	179.34 (17)
C5—N1—C1—C2	−1.6 (2)	C2—C3—C4—C5	−1.5 (3)
Cu1—N1—C1—C2	172.38 (13)	C7—C3—C4—C5	177.2 (2)
C5—N1—C1—C6	179.36 (16)	O1—C9—C10—C12	169.3 (2)
Cu1—N1—C1—C6	−6.7 (2)	O2—C9—C10—C12	−10.8 (3)
N1—C1—C2—C3	−0.7 (3)	O1—C9—C10—C11	−10.9 (3)
C6—C1—C2—C3	178.31 (18)	O2—C9—C10—C11	169.0 (2)
C1—N1—C5—C4	2.3 (2)	O1—C9—O2—Cu1	8.1 (2)
Cu1—N1—C5—C4	−171.75 (12)	C10—C9—O2—Cu1	−171.85 (13)
C1—N1—C5—C8	−177.78 (16)	N1^i^—Cu1—O2—C9	86.87 (13)
Cu1—N1—C5—C8	8.2 (2)	N1—Cu1—O2—C9	−93.13 (13)

Symmetry code: (i) −*x*+1, −*y*+1, −*z*+1.

### Hydrogen-bond geometry (Å, º)

**Table:**

*D*—H···*A*	*D*—H	H···*A*	*D*···*A*	*D*—H···*A*
C4—H4···O1^ii^	0.93	2.45	3.338 (2)	161
C6—H6*A*···O1	0.96	2.49	3.369 (3)	153
C6—H6*C*···O2^i^	0.96	2.48	3.139 (3)	126
C8—H8*A*···O1^i^	0.96	2.49	3.357 (3)	150
C8—H8*C*···O2	0.96	2.51	3.124 (2)	122

Symmetry codes: (i) −*x*+1, −*y*+1, −*z*+1; (ii) *x*+1, *y*, *z*.
